# Catheter ablation in children and young adults: is there an additional benefit from remote magnetic navigation?

**DOI:** 10.1007/s12471-013-0408-9

**Published:** 2013-04-18

**Authors:** R. W. Roudijk, M. Gujic, I. Suman-Horduna, P. Marchese, S. Ernst

**Affiliations:** 1 Department of Cardiology, UMC St. Radboud, Pegasusplaats 202, 6525 JK Nijmegen, the Netherlands; 2 Department of Cardiology, Royal Brompton and Harefield NHS Foundation Trust, London, United Kingdom, Sydney Street, London, SW3 6NP UK

**Keywords:** Catheter ablation, Cardiac arrhythmia, Young adults, Remote magnetic navigation, Congenital heart disease

## Abstract

**Purpose:**

Although rare, children and young adults can suffer from significant cardiac arrhythmia, especially in the context of congenital malformations and after cardiac surgery.

**Methods:**

A total of 62 patients (32 female, median age 20 years) underwent an invasive electrophysiology study between 2008–2011: half had normal cardiac anatomy, whereas the remaining patients had various types of congenital heart disease. All patients were treated using either conventional techniques (CVN) or remote magnetic navigation (RMN).

**Results:**

Patients treated with the RMN system differed substantially from patients in the CVN group with respect to presence of congenital heart disease (67 % vs. 37 %), previous cardiac surgery (59 % vs. 20 %) or failed previous conventional ablation (22 % vs. 9 %), respectively. Although these more complex arrhythmias resulted in longer median procedure duration (180 vs. 130 min, *p* = 0.034), the median overall fluoroscopy exposure in the RMN group was significantly lower (4.1 vs. 5.2 min, *p* = 0.020). Clinical outcome was comparable in both groups without complications caused by the ablation.

**Conclusions:**

Catheter ablation using remote magnetic navigation is safe and feasible in children and young adults and is especially valuable in patients with abnormal cardiac morphologies. RMN resulted in significantly lower radiation exposure compared with the conventional technique.

## Introduction

The prognosis of patients with congenital heart disease (CHD) has improved in recent decades [1]. Congenital heart surgery has made substantial progress so most of these children now survive and have become a large heterogeneous group of young adults with corrected heart anatomy [2–4]. Causative factors of arrhythmias in patients with CHD are: complex anatomy, scar and surgical patch material related re-entry [5, 6] accessory pathways, haemodynamic differences and atrial or ventricular enlargement [7]. Although the majority of patients with CHD and arrhythmias are treated with antiarrhythmic drugs, ablation therapy is an effective and curatively intended treatment option.

Especially in young patients it is very important to reduce the amount of radiation from fluoroscopy during electrophysiology procedures. These patients have a longer life expectancy, are more susceptible to radiation and are more likely to be exposed to more interventional procedures in the future [8]. The risk of radiation effects and radiation-induced cancer is significantly higher in young patients than in adult patients [9]. Minimising the radiation dose will reduce the late effects of radiation exposure [10, 11].

Precision and accurate navigation are essential to perform successful ablations in CHD patients and children, which can be achieved by using advanced technologies such as 3D electroanatomical mapping systems [12], image integration from CT or MRI and remote navigation by magnetic navigation [13]. We report on a single-operator experience using various techniques including magnetic navigation to treat symptomatic patients up to the age of 25 years.

## Methods

### Study design

This is an observational study of ablation procedures performed at the Royal Brompton Hospital under the direct or supervised care of a single consultant cardiologist (SE) during the period from October 2007 until October 2011. The data were exported from the local database (Lotus Notes) by the Quality and Safety Office and analysed retrospectively.

### Patient cohort

A total of 63 patients were referred to the electrophysiology (EP) department for ablation therapy because of symptomatic cardiac arrhythmias; one patient was excluded from analysis because no arrhythmia was induced during EP study. We included 62 patients who were not older than 25 at the date of the index ablation procedure. Treatment with either conventional or remote magnetic navigation was based on the type of arrhythmia and cardiovascular anatomy. Patients with pacemakers were not excluded from the remote magnetic navigation group.

### Electrophysiology procedure

Before the ablation a resting 12-lead ECG and laboratory tests were performed. Informed consent was obtained prior to the procedure. The procedures were performed either under local or general anaesthesia using intravenous sedation. The blood pressure of the patients was continuously monitored using radial or brachial arterial lines. Vascular access was gained via the femoral veins and arteries or, if necessary, via the subclavian or jugular veins. All patients underwent a post-ablation transthoracic echocardiogram to exclude pericardial effusion or damage to valvular structures.

### Pre-ablation imaging

In the presence of CHD or when cardiac abnormalities were suspected, the patients underwent a cardiac imaging study to acquire a 3D cardiac roadmap for image integration into the 3D electroanatomical mapping system (CARTO, Biosense Webster). Preference was given to a non-contrast cardiovascular magnetic resonance (CMR), but in case of an implanted device, a contrast cardiac computed tomography (CT) was performed.

### Conventional guided navigation

Conventional navigation (CVN) techniques are based on the electrical information provided by multiple diagnostic catheters and a manually steered ablation catheter. As an additional option, the electroanatomical mapping system CARTO (Biosense Webster, Brussels Belgium) was used when necessary [13].

### Remote magnetic navigation

The remote magnetic navigation (RMN) system (Stereotaxis Inc.) uses two permanent magnets to steer the magnetic tip of a floppy ablation catheter. The operator is able to change the directions of the magnetic field (0.08 Tesla) by changing the orientation of the outer magnetic field. Using a mechanical drive (Cardiodrive, Stereotaxis Inc) the magnetic catheter can be pulled back or pushed forward, thereby allowing the operator to perform the procedure from inside the control room [14]. The tip of the catheter is very soft and therefore is unlikely to perforate or damage the vessel and heart walls.

### Data collection and analysis

The data were analysed in subgroups based on navigation techniques, presence of CHD, age and the specific arrhythmia. The variables were chosen to describe the results and effects of the ablations. Total procedure time, total fluoroscopy time and radiofrequency (RF) time are presented as median values and their first and third quartiles. Total procedure time was defined as the time in minutes from the venous puncture to the removal of the catheter sheaths. The acute success of the procedure was measured using pacing protocols performed after ablation during the waiting time period. If no tachycardia could be induced 30 minutes after ablation, the procedure was acutely successful. Long-term effects of the procedure were measured using follow-up results from an outpatient clinic appointment: success was defined as a sinus rhythm on ECG or 24-hour Holter and symptom-free follow-up period of 6 months.

### Statistics

The results were analysed using an intention-to-treat protocol. SPSS version 17.0 (SPSS Inc., Chicago, IL) was used to perform statistical analysis. Qualitative variables were presented as percentages and comparisons were done using Pearson’s chi-squared test or Fisher’s exact test. Quantitative variables were presented as mean (SD) or median (IQR) and comparisons were done using the independent two-sample Student’s *t*-test or the two-sample Wilcoxon rank-sum test. A P-value of less than 0.05 was considered statistically significant.

## Results

### Population (Table 1, Figs. 1 and 2)

**Table 1:** Patient demographics and RMN vs. CVN procedure results

Parameters	All patients	RMN	CVN	P Value
N	62	27	35	0.310
Median age (1–3 QR)	19.7 (3.9)	21 (18–22)	20 (17–23)	0.976
Gender: female (N, %)	32 (51.6 %)	15 (56 %)	17 (49 %)	0.585
Congenital HD (N, %)	31 (50 %)	18 (67 %)	13 (37 %)	0.021^*^
Implanted pacemaker (N, %)	5 (8.1 %)	4 (15 %)	1 (3 %)	0.086
Previous surgery (N, %)	23 (37 %)	16 (59 %)	7 (20 %)	0.002^*^
Previous ablation (N, %)	9 (14.5 %)	6 (22 %)	3 (9 %)	0.130
Atrial tachycardia (N, %)	14 (22.6 %)	6 (22 %)	8 (23 %)	0.953
AVRT (N, %)	22 (35.5 %)	6 (22 %)	16 (46 %)	0.055
AVNRT (N, %)	14 (22.6 %)	6 (22 %)	8 (23 %)	0.953
Ventricular extrasystoles (N, %)	5 (8.1 %)	4 (15 %)	1 (3 %)	0.086
Ventricular tachycardia (N, %)	4 (6.5 %)	3 (11 %)	1 (3 %)	0.190
Atrial fibrillation (N, %)	1 (1.6 %)	1 (4 %)	0 (0 %)	0.251
Atrial flutter (N, %)	2 (3.2 %)	1 (4 %)	1 (3 %)	0.852
Pre-abation imaging (N)	19 (31 %)	7 CMR, 5 CT	5 CMR, 2 CT	0.101
Total procedure time (min, 1–3 IQR)	152 (98, 214)	180 (133.0–240.0)	130 (94.5–192.5)	0.034^*^
Fluoroscopy time (min, 1–3 IQR)	4.25 (2.5, 10.2)	4.1 (1.5–5.85)	5.2 (3.2–11.1)	0.020^*^
RF time (min, 1–3 IQR)	10.4 (5.5, 18.4)	11.7 (7.5–23.5)	10.0 (4.7–13.6)	0.094
General anaesthesia (N, %)	35 (56.5 %)	18 (67 %)	17 (49 %)	0.154
Acute success	91.9 %	93 %	91 %	0.867
Arrhythmia-free follow-up	67 %	65 %	69 %	0.765

Table with the demographics of the patient population and the procedure results in the remote magnetic navigation (RMN) and conventional navigation (CVN) group. The p-values show the differences between the two groups. Congenital HD: congenital heart disease; previous surgery: previous heart surgery for congenital or structural heart disease; *AVRT* atrioventricular reentrant tachycardia, *AVNRT* atrioventricular nodal reentrant tachycardia, *CMR* cardiac magnetic resonance imaging, *CT* computed tomography scan, *IQR* interquartile range; min: minutes, *RF* radiofrequency; acute success: successful procedure; follow-up: percentage free of arrhythmia

^*^Statistically significant

**Fig. 1:**
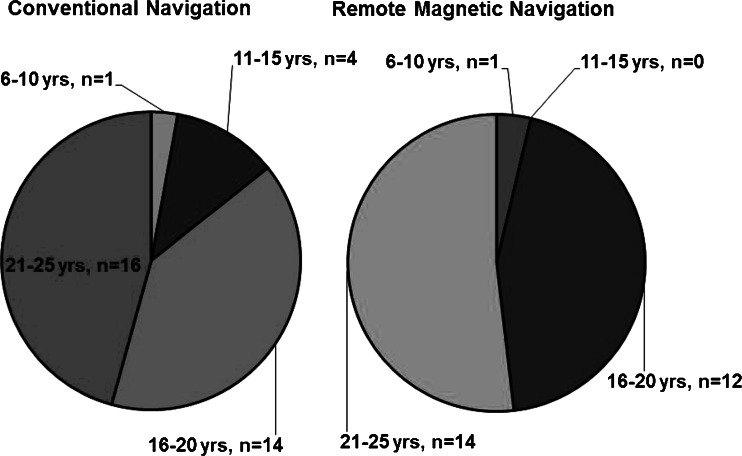
Age distribution. Pie chart of the age distribution of both patient groups: Left panel depicts the conventionally treated group (CNV), while the right panel shows the remote magnetic navigation (RMN) cohort

**Fig. 2:**
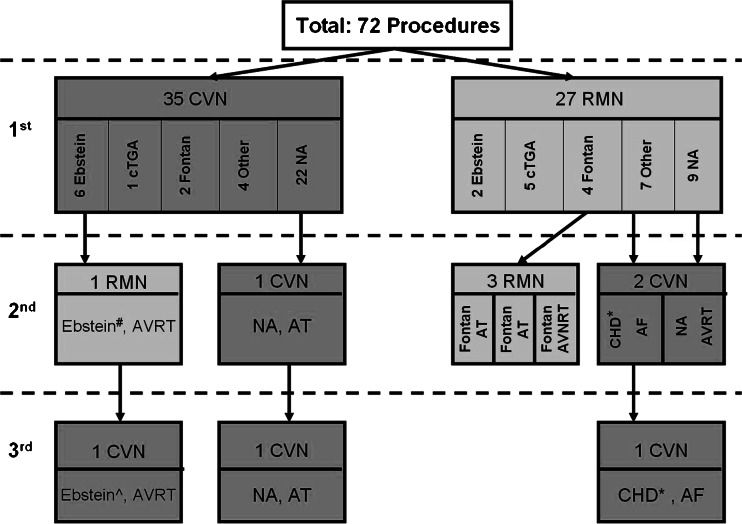
Flowchart of all ablation procedures. Flowchart of all 72 procedures in a total of 62 patients, with the majority (*N* = 55) having only one procedure. Four patients had a second procedure and 3 patients had a second and a third procedure. Abbreviations: RMN: remote magnetic navigation. CVN: conventional navigation, Ebstein: Ebstein’s anomaly, Fontan: Fontan-like circulation, NA: normal cardiac anatomy, cTGA: corrected transposition of the arteries, CHD: congenital heart disease, AT: atrial tachycardia, AVRT atrioventricular reentrant tachycardia, AVNRT: atrioventricular nodal reentrant tachycardia, *: Congenital mitral valve cleft with previous mitral valve repair and replacement, ^#^: Posterior pathway and an epicardial located accessory pathway, ^: Left posterior lateral accessory pathway

The median age of all 62 patients undergoing invasive EP procedures was 20 years (IQR: 18, 23) and 52 % were female. Two patients were younger than 10 years, four patients aged from 11–15 years, 26 patients aged from 16–20 years and 30 patients aged from 21–25 years. Patients were treated in the index procedure using either CVN (*n* = 35 patients) or RMN (*n* = 27 patients). CHD was present in 31 patients and five patients had an implanted pacemaker. More than one-third of the patients had undergone previous heart surgery for congenital or structural heart disease. The cohort includes patients after Mustard operation (*n* = 3 patients) or an atrial switch (*n* = 3 patients) for transposition of the great arteries, Fontan operation (*n* = 6), Ebstein’s anomaly (*n* = 8), ventricular septal defect (*n* = 4) or tetralogy of Fallot (*n* = 4). Nine patients had a failed previous CVN ablation in a different hospital.

The median age of patients in the RMN subgroup (*n* = 27) was 21 years (18, 22) and the median age in the CVN subgroup (*n* = 35) was 20 (17, 23). There were statistically significantly more patients with CHD (*p* = 0.021) and previous cardiac surgery (p = 0.002) in the RMN group compared with the CVN group. All patients’ baseline characteristics are listed in Table 1.

### Procedure parameters: RMN versus CVN (Table 2, Fig. 3)

**Table 2:** Procedure results in congenital heart disease: RMN vs. CVN

Parameters	RMN	CVN	P-value
N	18	13	0.369
Ebstein’s anomaly (N, %)	2 (11 %)	6 (46 %)	0.028^*^
Tetralogy of Fallot (N, %)	2 (11 %)	2 (15 %)	0.726
Corrected TGA (N, %)	5 (28 %)	1 (8 %)	0.164
Post Fontan procedure (N, %)	4 (22 %)	2 (15 %)	0.634
Ventricular septal defect (N, %)	2 (11 %)	2 (15 %)	0.726
Previous cardiac surgery (N, %)	16 (89 %)	7 (54 %)	0.030^*^
Previous ablation (N, %)	5 (28 %)	2 (15 %)	0.423
Targeted arrhythmia			
Atrial tachycardia (N, %)	6 (33 %)	7 (54 %)	0.253
Ventricular tachycardia (N, %)	3 (17 %)	1 (8 %)	0.462
Ventricular extrasystoles (N, %)	4 (22 %)	0 (0 %)	0.069
AVNRT (N, %)	2 (11 %)	0 (0 %)	0.214
AVRT (N, %)	1 (6 %)	5 (38 %)	0.022^*^
Atrial fibrillation (N, %)	1 (6 %)	0 (0 %)	0.388
Atrial flutter (N, %)	1 (6 %)	0 (0 %)	0.388
Procedure parameters			
General anaesthesia (N, %)	15 (83 %)	10 (77 %)	0.661
Pre-ablation imaging (N)	6 CMR, 3 CT	1 CMR	0.453
Median procedure time (min, 1–3 IQR)	186 (154, 264)	203 (130, 223)	0.674
Median fluoroscopy time (min, 1–3 IQR)	3.7 (2.3, 6.2)	4.1 (3.3, 11)	0.123
Median radiofrequency time (min, 1–3 IQR)	18.2 (9.4, 24.6)	12.2 (11, 22.4)	0.603
Acute success	94 %	100 %	0.395
Arrhythmia-free follow-up	67 %	62 %	0.873
Redo ablation (N,%)	4 (22 %)	1 (8 %)	0.286

Table with the procedure results of the subgroup analysis of patients with congenital heart disease. *SD* standard deviation, *TGA* transposition of the great arteries, *AVRT* atrioventricular reentrant tachycardia, *AVNRT* atrioventricular nodal reentrant tachycardia, *CMR* cardiac magnetic resonance imaging, *CT* computed tomography scan; min: minutes, *IQR* interquartile range, arrhythmia-free follow-up: six-month follow-up

^*^statistically significant

**Fig. 3:**
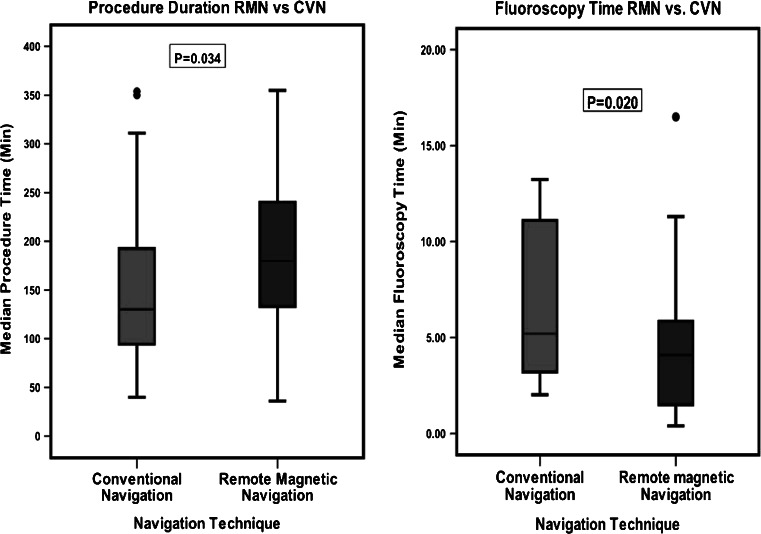
Boxplot comparison of RMN vs. CVN. Comparison of procedure duration and overall fluoroscopy exposure times during the index procedure for all patients

The median procedure time was significantly longer in the RMN group compared with the CVN group, 180 min (133, 240) vs. 130 min (94.5, 192,5) respectively (*p* = 0.034). The median fluoroscopy time in the RMN group was statistically significantly lower compared with the CVN group, 4.1 min (1.5, 5.9) vs. 5.2 min (3.2, 11.1) respectively (*p* = 0.020). Both groups had similar median RF times and success rate. CVN had no acute success in 3 patients with normal cardiac anatomy: 1 patient with atrioventricular reentrant tachycardia (AVRT), 1 patient with ventricular extrasystoles and 1 with atrial tachycardia. The RMN group had no acute success in 1 patient with ventricular tachycardia after atrial switch surgery for transposition of the great arteries and 1 patient with normal cardiac anatomy and AVRT. After six-month follow-up there was no difference in arrhythmia-free patients between the RMN and the CVN group (65 % vs 69 % *p* = 0.765).

### RMN versus CVN: Congenital heart disease subgroup (Table 2, Fig. 4)

**Fig. 4:**
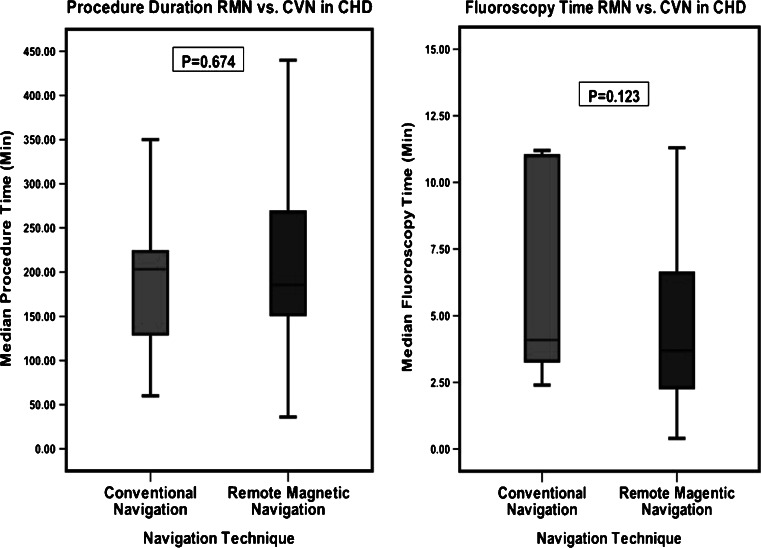
Boxplot comparison of RMN vs. CVN in congenital heart disease. Comparison of procedure duration and overall fluoroscopy exposure times during the index procedure for all patients with congenital heart disease (CHD)

The subgroup analysis of CHD patients compared the patients treated with RMN (*n* = 18) and the patients treated with CVN (*n* = 13) techniques. The CVN group consisted of significantly more Ebstein’s anomaly patients (6 versus 2 patients, *p* = 0.028) and patients with AVRT (1 versus 5 patients, *p* = 0.022). Previous cardiac surgery for CHD was present in 89 % of the patient treated with RMN, a significant difference compared with the CVN group (*p* = 0.030). There was no significant difference in procedure and fluoroscopy time between the groups. The median procedure time in the RMN group was 186 min (154, 264) and the median procedure time in the CVN group was 203 min (130, 223). The median fluoroscopy time was 3.7 min (2.3, 6.2) compared with 4.1 min (3.3, 11.0) in the CVN group. Both groups had similar acute success rates and comparable six-month follow-up results.

### Complications

Post-ablation transthoracic echocardiography showed no pericardial effusion, tamponade or damage to the aorta and mitral valve. There were no other major complications, except for one patient, who developed a haemothorax after positioning of a central jugular venous catheter used for anaesthesia.

## Discussion

The results of our retrospective study show that catheter ablation of arrhythmias in children and young adults is effective and safe. RMV is highly effective even in young patients and even in the presence of CHD. In the context of these young and more complex patients, they are more likely to relapse into tachycardia in the future [2]. Triedman et al. report a recurrence rate of atrial tachycardia of 53 % in CHD patients after 3 years [15]. In our cohort, only 11.3 % of the patients needed a redo ablation, which seems acceptable regarding the type of patients included in this study. To summarise, these young CHD patients have more complex tachycardia substrates, a higher risk for redo ablation, a higher radiation exposure and a lower success rate of ablation.

### Remote navigation versus conventional ablation techniques

There are only few reports in the literature about catheter ablations with RMN in children and young adults [15, 16]. However the RMN system has the advantage of the floppy and soft catheter tip that is less likely to perforate the thin wall of the heart or blood vessel. The RMN system has been previously demonstrated to be effective in the treatment of cardiac arrhythmias in an adult CHD patient population [16, 17].

### Reduction of radiation exposure in children and young adults

It is essential to reduce radiation exposure in general as much as possible [18]. The short- and late-term effects of fluoroscopy exposure are more distinct in children and young adults [8]. The overall fluoroscopy exposure during catheter ablation is generally higher in CHD patients [2, 16]. The RMN system has been reported to significantly reduce fluoroscopy exposure during catheter ablation in various patient cohorts. [19–21] Although we managed to successfully treat patients in our conventional group with very low exposure to radiation as compared with previously published reports [16, 17, 22], we could still demonstrate a significant reduction in exposure when using magnetic navigation although those patients were more complex to treat. The effect of reduction of radiation exposure by using remote navigation has been previously reported by other groups [23, 24] for normal morphology patients and also for CHD patients or young patients [16, 25]. The significant reduction in radiation exposure by using RMN in our study is similar compared with that of Schwagten et al. [16, 17], but the total fluoroscopy exposure in our CVN group was already lower than their RMN group. This absolute difference in procedure and fluoroscopy time could be potentially explained by the more extensive use of image integration and 3D mapping in our study.

### Limitation in vascular access in paediatric patients

Our cohort mainly consisted of adolescents and young adults, while only about 10 % of patients were under the age of 15 years. This is mainly due to the local referral basis, but also reflects on the obvious limitations to vascular access in younger children. The diameter of the available ablation catheters for the RMN system is currently limited to 7 French; however, in the conventional group no 6 French catheters were used. This could impose a limitation for using remote navigation in even younger children.

### Complications

During ablation procedures in adults the overall complication risk is 3.8 %, with a risk for cardiac perforation of 1.3 % [26]. In our study, we had a comparably lower complication rate, with only one case of a haemothorax (1.6 %).

### Limitations

Considering the heterogeneity of CHD and arrhythmia substrates and the small numbers in the specific arrhythmia groups, all the subgroup analyses performed are difficult to interpret and we have therefore limited this to the presence of CHD. A multicentre prospective randomised clinical trial would be needed to obtain more reliable data about success and complication rates of catheter ablation in children and young adults using advanced technology.

## Conclusion

Catheter ablation using remote magnetic navigation is safe and feasible in children and young adults. In comparison to a conventionally treated similar group of patients by the same operator, RMN significantly lowered fluoroscopy exposure. The use of advanced technology including integration of pre-acquired 3D imaging and 3D electro-anatomical mapping was especially valuable in patients with complex congenital heart disease.
